# Dupilumab-associated ocular surface disease: presentation, management and long-term sequelae

**DOI:** 10.1038/s41433-020-01379-9

**Published:** 2021-01-28

**Authors:** Magdalena Z. Popiela, Ramez Barbara, Andrew M. J. Turnbull, Emma Corden, Beatriz Suarez Martinez-Falero, Daniel O’Driscoll, Michael R. Ardern-Jones, Parwez N. Hossain

**Affiliations:** 1 Eye Unit, Southampton General Hospital, Southampton, UK; 2 Eye Unit, St Bernard’s Hospital, Gibraltar, Gibraltar; 3 Bournemouth Eye Unit, Royal Bournemouth Hospital, Bournemouth, UK; 4 Department of Dermatology, Southampton General Hospital, Southampton, UK; 5 Clinical Experimental Sciences, University of Southampton, Southampton, UK

**Keywords:** Eye manifestations, Conjunctival diseases

## Abstract

**Objectives:**

To determine the presenting features of ocular surface disease in patients with atopic dermatitis (AD) treated with dupilumab at a tertiary, university hospital. To establish the need for treatment of dupilumab-associated ocular surface disease and report any long-term effects on the ocular surface.

**Methods:**

A retrospective analysis of consecutive patients treated with dupilumab for AD between January 2017 and August 2019 was undertaken. Data were collected on demographics, incidence and type of ocular disease features, natural history and treatment.

**Results:**

A total of 50% (14/28) patients developed ocular symptoms with a mean time of onset of 6.75 (±6.1) weeks from starting dupilumab. Of these, 69% (9/13) were diagnosed with conjunctivitis associated with cicatrisation in two patients and periorbital skin changes in four. Of these nine, four had prior history of atopic keratoconjunctivitis. All were treated with topical steroids; two required additional ciclosporin drops. In all, 67% (6/9) patients went on to have on-going ocular inflammation requiring maintenance drops at a mean of 16 (±6.9) months of follow-up. All patients had improvement in their AD severity; only one patient discontinued dupilumab due to ocular side effects.

**Conclusion:**

The rate of dupilumab-associated ocular surface disease was 32%. Periorbital skin changes and conjunctival cicatrisation were noted in association with conjunctivitis. Ocular surface disease improved on topical steroids and ciclosporin but 67% of patients needed on-going treatment. Close liaison with an ophthalmologist should be considered in those patients who develop conjunctivitis or have a past history of severe ocular surface disease.

## Introduction

Dupilumab is the first biologic approved for use in treatment of moderate to severe atopic dermatitis (AD) [1–5]. Dupilumab targets interleukin (IL)-4 receptor blocking IL-4 and IL-13 signalling pathway. It has also shown promise in treatment of asthma, chronic rhinosinusitis with nasal polyposis and eosinophilic oesophagitis [1, 2].

Dupilumab has been found to significantly improve signs and symptoms of AD and is considered a safe treatment for long-term use [6]. Conjunctivitis is a known side effect with rates in initial trials varying between 8.6% (CHRONOS) and 28 % (LIBERTY AD CAFÉ). Conjunctivitis is considered mild and self-limiting in the majority of cases, with fewer than 0.5% of patients suffering from a severe form necessitating drug cessation [2].

Current International Eczema Council recommendations allow for dupilumab to be started in patients with prior ocular surface disease and to continue dupilumab in the event of conjunctivitis occurring [7]. Prior history of ocular surface disease and more severe AD are considered risk factors for the development of dupilumab-associated conjunctivitis [8, 9].

Since the start of clinical use, apart from self-limiting conjunctivitis other ocular features have been described: severe follicular conjunctivitis [10, 11], limbal nodules [4, 10], blepharoconjunctivitis [10–14], cicatricial ectropion [15, 16], keratitis [10] and dry eyes [17]. This has led to the use of the term dupilumab-associated ocular surface disease to encompass various clinical presentations [8, 9, 16]. A much higher incidence of ocular surface problems has been reported in clinical practice than in the initial drug trials—as high as 70% in one series [4]. No data are available on the longer-term effects of dupilumab on the ocular surface. Our study aimed to establish local rates of dupilumab-associated ocular surface disease, describe its features, establish the need for treatment and identify any long-term sequelae.

## Methods

The Health Research Authority and Health and Care Research Wales approval was granted in November 2019 (19/HRA/5882) for a retrospective case review of all patients developing dupilumab-associated eye disease at Southampton General Hospital—a large tertiary referral centre in the United Kingdom.

Patients prescribed dupilumab between January 2017 and August 2019 were identified from the severe AD clinic in the dermatology department. Their details were checked in the electronic patient records to identify those who attended ophthalmology clinics. Data for patients who developed eye symptoms were collected retrospectively in February 2020 to allow for at least 6 months of ophthalmic follow-up. Presenting ocular symptoms, signs on initial review, treatment, visual acuity at first and last follow-up and any side effects of topical therapy were noted. Long-term use of treatment was recorded. Eczema Area and Severity Index (EASI) scores were recorded at pre-treatment and at 16 weeks post dupilumab initiation, as well as the percentage of patients reaching 50 and 75% improvement in EASI at 16 weeks (EASI 50 and EASI 75, respectively).

### Statistics

Because of only a few studies for reference, the study is classed as an exploratory research study. Data were analysed using descriptive statistics using The SAMPL Guidelines as reference (https://www.equator-network.org/wp-content/uploads/2013/03/SAMPL-Guidelines-3-13-13.pdf) with the statistical tools within Microsoft Excel 2014 (Microsoft Corp., Redmond, WA, USA). Logistic regression and Mann–Whitney *U* test were used to test for differences in demographic data between patients with and without ocular symptoms. Statistical significance was defined with a *P* value of equal to or less than 0.05. All statistical analyses were performed using SPSS software (version 20.0, IBM Corp, Armonk, NY, USA).

## Results

A total of 28 patients were prescribed dupilumab for AD between January 2017 and August 2019. Fourteen patients were referred to the ophthalmology department with symptoms of bilateral eye redness, soreness, itching and epiphora with a mean time of onset of 6.75 (±6.1) weeks from starting dupilumab. One patient was lost to follow-up with the remaining 13 cases seen in the ocular surface clinic under the care of the ophthalmology department. There were no significant differences in demographic characteristics of patients treated with dupilumab who developed ocular symptoms and those who did not develop ocular symptoms [Table 1].

**Table 1:** Demographic data of patients with and without ocular symptoms while on dupilumab.

	Patients with ocular symptoms while on dupilumab	Patients without ocular symptoms	*P* value
Age ± SD (years)	38.5 ± 12.1	33.5 ± 13.4	0.194
Gender (F: female, M: male)	7 F, 7 M	5 F, 9 M	0.352
Race	12 Caucasians, 1 of Asian descent, 1 not recorded	11 Caucasians, 1 of Asian descent, 2 not recorded	0.355

Four patients seen in the ophthalmology department showed reduced tear break up time (TBUT < 10 seconds) with meibomian gland disease and were diagnosed as having evaporative dry eyes without signs of conjunctivitis. They were prescribed lubricating eye drops and given advice on lid hygiene. One patient received a course of Maxitrol ointment for eyelid disease. They were excluded from further analysis.

**Fig. 1:**
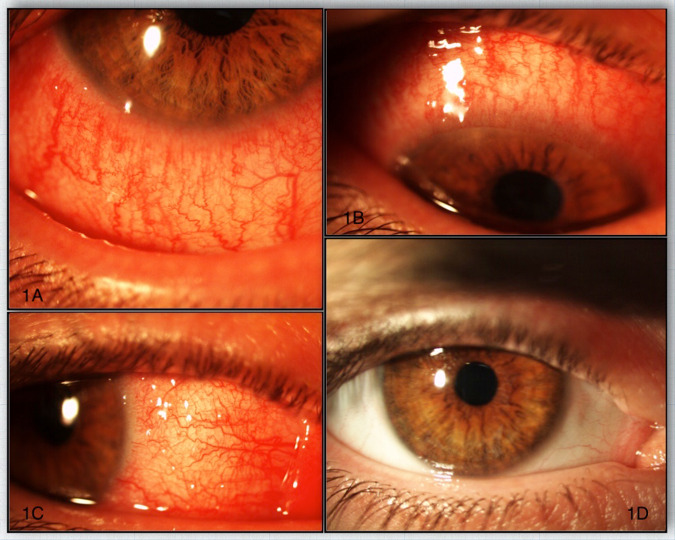
Acute presentation of dupilumab-associated conjunctivitis and its resolution with drops. **A**–**C** Patient with acute dupilumab-associated conjunctivitis. **D** Same patient 6 weeks post initiation of steroid therapy.

Nine patients showed features of conjunctivitis on initial presentation with bilateral marked conjunctival redness [Fig. 1]. Six patients displayed bilateral conjunctival papillary reaction and three had bilateral conjunctival follicular changes. Two patients showed limbal nodules similar in appearance to Trantas dots at initial visit [Table 2].

**Table 2:** Data on patients with dupilumab-associated ocular surface disease.

	Age, gender	Past ocular history	Skin response	Symptoms	Signs on presentation	Onset of symptoms (weeks)	Treatment	Follow-up (months)	Response to drops
1.	24, F	None	EASI 75	Bilateral painful eyes	Bilateral follicular conjunctivitis	16	FML and lubricants	20	FML OD
2	57, F	HSV keratitis	EASI 75	Bilateral itchy, red, watery eyes	MGD, papilla, conjunctival scarring left lower fornix	1	Prednisolone 0.5%	24	Developed leukoplakic lesion in lower fornix, steroid drops stopped after 12 months
3.	28, M	AKC	Improvement	Bilateral red, sore watery eyes	Bilateral limbal nodules, papillary conjunctivitis and lid erythema	4	Prednisolone 0.5%, Ikervis, Opatanol and lubricants	26	Prednisolone 0.5% OD
4.	43, F	AKC	EASI 75	Episodes of redness	Redness, chemosis, papillary changes	4	Prednisolone 0.5%, lubricants	16	Prednisolone 0.5% OD
5.	54, F	None	EASI 75	Bilateral sore, red, watery eyes	Bilateral follicular conjunctivitis, MGD, cicatricial changes in upper and lower fornix, cicatricial ectropion	12	Prednisolone 0.5%, Ikervis, Betnesol and Tacrolimus ointments to eye lids	16	Prednisolone 0.5% OD, Ikervis OD
6.	34, M	AKC	EASI 75	Bilateral sore, red, watery eyes	Bilateral limbal nodules, severe papillary conjunctivitis	8	Betnesol ointment	14	Resolution, 8 months use of steroids
7.	26, F	None	EASI 50 prior to stopping	Bilateral red eyes, loss of lashes	Bilateral follicular conjunctivitis, loss of lashes, periorbital dermatitis	2	Dexamethasone preservative free, lubricants	Lost to follow-up	Lost to follow-up
8.	43, M	None	EASI 50	Bilateral red sore eyes	Papillary changes, periocular redness, punctual stenosis	1	Prednisolone 0.5% and lubricants	6	Prednisolone 0.5% OD
9.	51, M	KC, AKC	EASI 50	Bilateral redness	MGD, mild redness and papillary reaction	Unspecified	Dexamethasone preservative free, lubricants	6	Resolution after 3 months

*F* female, *M* male, *AKC* atopic keratoconjunctivitis, *HSV* herpes simples virus, *KC* keratoconus, *FML* fluorometholone 0.1% drops, *Ikervis* ciclosporin 0.1% drops, *EASI 50* 50% improvement in Eczema Area and Severity Index score at 16 weeks, *EASI 75* 75% improvement in Eczema Area and Severity Index score at 16 weeks, *OD* once a day for drop frequency.

**Fig. 2:**
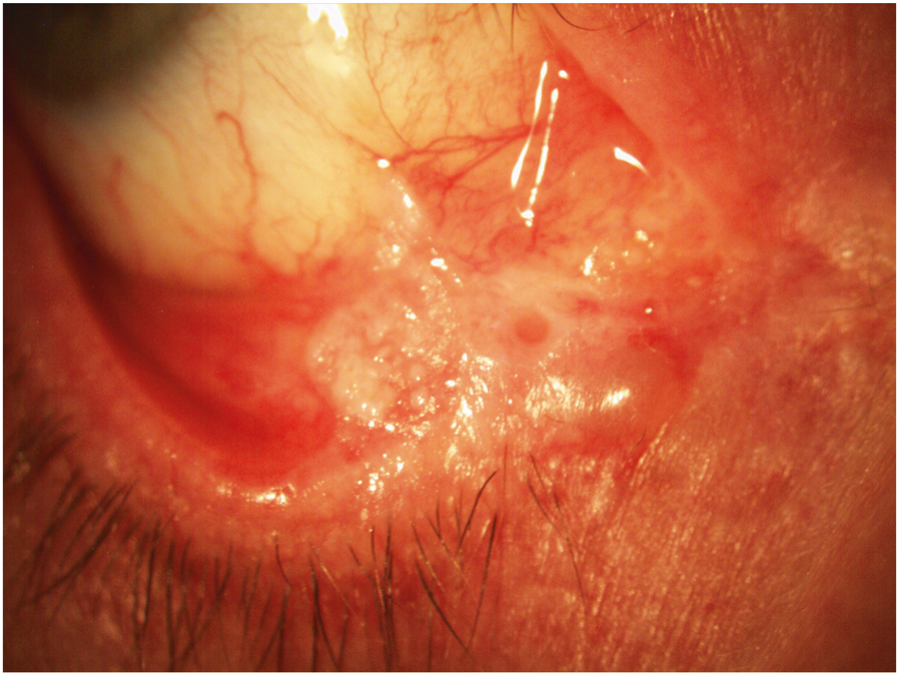
Conjunctival cicatrisation in patient on dupilumab. Patient on dupilumab with progressive cicatrisation and leukoplakic lesion in lower fornix.

Two female patients, who were sisters, had associated conjunctival cicatrisation in the lower fornix and one showed scarring in the superior tarsal conjunctiva on initial presentation. One of these patients went on to develop progressive cicatrisation and a leukoplakic lesion in the lower fornix, confirmed on biopsy as pre-cancerous actinic keratosis with dysplasia [Fig. 2].

Periocular changes with cicatrising ectropion, punctal stenosis and periocular dermatitis were noted in four patients in association with conjunctivitis.

Mean time to development of self-reported eye symptoms after the start of dupilumab therapy in the conjunctivitis group was 6 ± 5.5 weeks. Patients were seen in the ocular surface clinic on average 8 ± 5.2 weeks later. A total of 44.4% patients (4/9) had a past history of atopic keratoconjunctivitis (AKC) prior to receiving dupilumab. Their ocular surface disease was stable at the time of dupilumab initiation with subsequent development of new symptoms of conjunctivitis. None were receiving active ophthalmic treatment at the time of starting dupilumab.

Initial treatment in the eye clinic for dupilumab-associated conjunctivitis included topical steroids in all cases. Six patients received additional topical lubricant. Prednisolone 0.5% in either preservative free or preserved preparation was used in five patients, Fluorometholone 0.1% (FML®, Allergan) drops in one, dexamethasone 0.1% preservative free preparation in two and Betamethasone 0.1% (Betnesol, RPH Pharmaceuticals) ointment in one patient. Initial frequency was one drop four times a day for all drops and twice a day for the ointment. Out of nine patients presenting with conjunctivitis, seven had a good response to topical steroids alone [Fig. 1], two required additional topical ciclosporin 0.1% (Ikervis®, Santen Pharmaceuticals) to control symptoms. One patient was prescribed tacrolimus ointment to the eyelids by the dermatologist. Topical steroid frequency was tapered down according to response with initial aim to get all patients off drops where possible.

A total of 67% (6/9) patients were on maintenance topical steroid or steroid and ciclosporin combination at a mean of 16 ± 6.9 months follow-up. Maintenance steroid drops were the same, as used at the beginning of therapy with reduced frequency of application down to once per day [Table 2]. The remaining three out of the nine (33%) patients used topical steroid for 3–12 months with complete resolution of symptoms and signs of conjunctivitis. Two of six patients (33%) needing maintenance steroids had a prior history of AKC, which was quiescent at the time of starting dupilumab.

We have not noted any changes in visual acuity from first to last visit; mean visual acuity was recorded as 0.1 logMAR at both time points. No patients developed side effects due to prescribed drops. All patients had an improvement in their EASI scores, with a 76% mean improvement in EASI score from pre-treatment to 16 weeks follow-up in the conjunctivitis group. In all, 89% of patients achieved EASI 50 (50% improvement in EASI score) and 55.6% achieved EASI 75 (75% improvement in EASI score) [Table 3]. One patient required additional therapy with methotrexate and one with topical steroids; in both cases EASI scores have improved. Dupilumab was discontinued in one patient due to severe conjunctivitis with loss of eye-lashes (madarosis).

**Table 3:** Eczema Area and Severity Index (EASI) scores in patients with conjunctivitis.

Patient	EASI pre-treatment	EASI at week 16	EASI 50 achieved	EASI 75 achieved
1	9.2	1	Yes	Yes
2	12.5	1	Yes	Yes
3	4.9	2.5	No	No
4	16.8	2.4	Yes	Yes
5	51.4	8.6	Yes	Yes
6	17	1	Yes	Yes
7	26	12.9	Yes	No
8	24	7.5	Yes	No
9	5	2	Yes	No

*EASI 50* at least 50% reduction in Eczema Area and Severity Index score at 16 weeks, *EASI 75* at least 75% reduction in Eczema Area and Severity Index score at 16 weeks.

## Discussion

Conjunctivitis is a known side effect of dupilumab, particularly affecting patients with AD [1, 2]. The LIBERTY AD CAFÉ study reported the highest incidence of dupilumab-associated conjunctivitis amongst initial trials (28%), attributed to higher awareness of this side effect amongst investigators [1]. The CAFÉ trial reported conjunctivitis in 22.1% of patients, which was thought to be due to the high percentage of participants with severe AD and prior AKC, both of which are considered risk factors [8, 9].

In our series, we found a higher rate of dupilumab-associated conjunctivitis (32%) compared to landmark studies [2]. This is in keeping with other clinical reports of real-world outcomes. Dupilumab-associated ocular surface disease was reported in 43% and 70% by Nahum et al. and Ivert et al., respectively [5, 9]. These differences could be due to overall smaller numbers of patients included in the clinical reports compared to landmark studies [2, 4, 5, 10]. Alternatively, many patients with AD might have undiagnosed ocular surface disease that gets exacerbated by the use of dupilumab and only then is brought to the attention of treating ophthalmologists. With retrospective data collection we could not establish how many of our patients had pre-existing undiagnosed ocular surface disease. We know that 44.4% had confirmed prior history of allergic eye disease with half requiring long-term ocular treatment to control their symptoms after starting dupilumab. Furthermore, a large percentage of our patients (67%) needed an on-going therapy for their ocular surface disease with mean duration of follow-up of 16 (±6.9) months.

The aetiology of dupilumab-associated conjunctivitis is not fully understood but shares some common characteristics with atopic blepharoconjunctivitis and allergic eye disease—namely decrease in goblet cells, heightened OX40 ligand activity, eosinophilia and increased Demodex infestation due to changes in the ocular surface environment [10, 18–23]. Allergic blepharokeratoconjunctivitis is common in AD with 32–56% of patients affected [18, 22–24]. Previous history of AKC and more severe AD prior to dupilumab initiation have been implicated as risk factors for developing dupilumab-associated ocular surface disease [8, 9].

Akinlade et al. reported that 80% of patients had resolution of conjunctivitis while on dupilumab in clinical trials, but there was no mention of duration of symptoms, nor what therapy these patients received for treatment [2]. It also leaves 20% of cases with on-going ocular surface inflammation while on dupilumab. These findings support our theory that dupilumab-associated conjunctivitis might not be just a one off event in all affected eyes. The drug might alter the ocular surface flaring up the susceptibility to chronic blepharoconjunctivitis in patients with AD. Alternatively, a large percentage of patients with AD considered for dupilumab have previously undiagnosed ocular surface inflammation. Clinically, it is difficult to distinguish a blepharoconjunctivitis flare up from dupilumab-associated ocular surface disease as they share many similarities [2, 9–18]. In the series of Maudinet et al., 64% of patients had previously undiagnosed blepharoconjunctivitis when examined prior to starting dupilumab. Interestingly, lower rates of dupilumab-associated conjunctivitis were observed in patients seen and treated by the ophthalmologist prior to starting the drug than in patients not seen by ophthalmologist prior to dupilumab therapy (13% versus 25%, respectively).

Current practice in our unit is that the ophthalmologist reviews patients after they develop ocular symptoms while on dupilumab. This practice frequently means delay in being assessed, which in turn makes it difficult to be certain if ocular features are solely result of dupilumab or previously undiagnosed AKC. Also dupilumab is considered in patients with high predisposition for chronic ocular surface disease. More emphasis should be placed on enquiring about ocular symptoms in patients with AD prior to starting dupilumab. Close liaison with an ophthalmologist should be considered in symptomatic patients and those with a prior history of ocular surface disease prior to starting dupilumab plus those who develop conjunctivitis. Some consideration should also be given to examining all patients prior to starting dupilumab by an ophthalmologist.

In our series all patient presented with severe bulbar conjunctiva injection, associated more commonly with papillary response and all necessitated steroid therapy.

Maudinet et al. [10] reported features of dupilumab-associated conjunctivitis in keeping with our findings. In their series, 60% of patients presented with mild injection and papillary conjunctivitis and were treated with topical lubricants, whereas the remaining 40% had severe follicular conjunctivitis requiring topical steroid treatment. In our cohort we did not find that differentiating between papillary and follicular presentation was clinically relevant.

Importantly, two patients in our study had cicatrising changes present in the lower fornix and subtarsal conjunctiva at presentation. These two patients were sisters, perhaps implicating a genetic predisposition for developing a more severe immune response to dupilumab. This could also be a result of their AD alone and/or due to the presence of chronic ocular inflammation. Again neither had a history of AKC nor symptoms of chronic ocular surface problems requiring ophthalmic care prior to starting dupilumab. Another explanation for the severity of ocular surface inflammatory features in these two cases could be related to a delay in ophthalmic review between developing dupilumab-related ocular symptoms and being seen in the ocular surface clinic. In one case this time delay was 5 months (patient 5, see Table 2).

One of the patients affected by cicatrisation went on to develop a progressive leukoplakic lesion in the lower conjunctival fornix, which was confirmed histologically to be actinic keratosis with dysplasia—a precancerous lesion. This was thought to be a result of life-long immunosuppression and not solely a result of dupilumab use. This patient was continued on dupilumab due to good dermatological response. Further excisional biopsy with postoperative adjuvant treatment with Interferon alpha-2b drops was planned at last follow-up.

One patient (3.5%) in our series had to discontinue dupilumab due to severe conjunctivitis and loss of lashes (madarosis). Madarosis has not been described before and represents an extreme reaction to dupilumab [2, 5, 10–14]. To our knowledge, there are two other case reports of conjunctival cicatrisation associated with dupilumab use [12, 14]. Associated cicatrising ectropion, periocular dermatitis and punctal stenosis have been described more commonly [10–15].

Dupilumab is also known to increase the risk of orofacial herpes simplex infection recurrence [2, 5]. Herpes simplex virus (HSV) uveitis while on dupilumab has been described with no reports of HSV keratitis [5]. One patient in our series had previous history of HSV keratitis but did not develop any self-reported herpetic eye disease flare-ups whilst on dupilumab. No patients in our series were swabbed for the presence of HSV or any other infective pathogens at the time of their acute presentation. Infective aetiology cannot be ruled out for certain as a cause of their acute ‘dupilumab-associated’ conjunctivitis presentation. However, no patients demonstrated any of the typical features of herpes simplex or adenoviral keratitis or the clinical course suggestive of infective disease. We consider HSV, adenovirus, Chlamydia or bacteria as causative agents to be unlikely.

All of our patients had improvement in ocular symptoms on topical steroids, which were the mainstay of the initial treatment [25]. Topical steroids alone were sufficient at reversing signs and symptoms of acute conjunctivitis in 89% of patients. Two patients required additional ciclosporin drops to control their symptoms. Topical preparations of ciclosporin have been successfully used in treatment of ocular surface disease for many years. Ciclosporin is a calcineurin inhibitor that inhibits T-cell-mediated immune responses and has been shown to increase goblet cell numbers [26]. Dupilumab targets IL-13 action and secondary depletion of goblet cells and mucin production in the conjunctiva could be responsible for dupilumab-associated conjunctivitis [2–5]. Ciclosporin might be particularly useful in treatment of dupilumab-associated ocular surface disease as it could reverse some of the local immunological actions of dupilumab. Use of Ikervis (ciclosporin 0.1%) drops in dupilumab-associated ocular surface disease remains off label since Ikervis is approved for use in dry eyes [27]. In our series, Ikervis drops used twice a day were added to topical steroids with good clinical outcomes. Use of tacrolimus [4, 8] and pimecrolimus [28] ointments to the eye lids has also been reported to improve dupilumab-associated ocular surface disease in other case series. No patient in our cohort developed side effects due to topical therapy during the follow-up and vision remained excellent. All had improvement in EASI scores.

With the retrospective nature of our study and small sample it is impossible to say how many patients had pre-existing undiagnosed AKC or whether dupilumab sensitised their eyes to become chronically inflamed. Larger long-term prospective studies would be needed to answer this question. We also lack data on the exact duration of the acute dupilumab-associated conjunctivitis due to retrospective data collection. In our series of patients, those not requiring maintenance steroid drops used topical steroids between 3 and 12 months.

In summary, dupilumab is used in patients who have a strong predisposition to chronic ocular surface problems. In our series, 32% were affected by acutely presenting dupilumab-associated ocular surface disease, which responded well to topical steroids with or without adjunctive topical ciclosporin. Dupilumab-associated conjunctivitis can lead to conjunctival cicatrisation and be associated with periocular skin changes, punctal stenosis and madarosis, making dupilumab-associated ocular surface disease a more useful, catch-all term. Dupilumab may unmask a predisposition to chronic ocular inflammation and a significant number of patients need long-term topical immunosuppressive therapy. Close liaison with an ophthalmologist should be sought in patients who develop conjunctivitis, symptomatic patients and those with a past history of ocular surface disease prior to starting the drug.

### Summary

#### What was known before

- Conjunctivitis is a known side effect of dupilumab therapy in patients with atopic dermatitis.
- Features vary from self-limiting conjunctivitis to periocular dermatitis making dupilumab-associated ocular surface disease a more descriptive term.
- Topical steroids and ciclosporin are mainstay of therapy for dupilumab-associated ocular surface disease.

#### What this study adds

- Dupilumab conjunctivitis can lead to conjunctival cicatrisation and madarosis. Periocular changes are frequently present in conjunction with conjunctivitis.
- After the acute episode of dupilumab-associated conjunctivitis, large proportion of patients need long-term drops to control on-going ocular surface inflammation.
- Close liaison with an ophthalmologist should be considered in those patients who develop conjunctivitis, are symptomatic or have a past history of ocular surface disease prior to starting dupilumab.

## References

[1] de Bruin-Weller M, Thaçi D, Smith CH, Reich K, Cork MJ, Radin A (2018). Dupilumab with concomitant topical corticosteroid treatment in adults with atopic dermatitis with an inadequate response or intolerance to ciclosporin A or when this treatment is medically inadvisable: a placebo-controlled, randomized phase III clinical trial (LIBERTY AD CAFÉ). Br J Dermatol.

[2] Akinlade B, Guttman-Yassky E, de Bruin-Weller M, Simpson EL, Blauvelt A, Cork MJ (2019). Conjunctivitis in dupilumab clinical trials. Br J Dermatol.

[3] Chu CY (2019). Keeping an eye on the ocular problems in dupilumab clinical trials. Br J Dermatol.

[4] Wollenberg A, Ariens L, Thurau S, van Luijk C, Seegräber M, de Bruin-Weller M (2018). Conjunctivitis occurring in atopic dermatitis patients treated with dupilumab-clinical characteristics and treatment. J Allergy Clin Immunol Pract.

[5] Ivert LU, Wahlgren CF, Ivert L, Lundqvist M, Bradley M (2019). Eye complications during dupilumab treatment for severe atopic dermatitis. Acta Derm Venereol.

[6] Deleuran M, Thaçi D, Beck LA, de Bruin-Weller M, Blauvelt A, Forman S (2020). Dupilumab shows long-term safety and efficacy in patients with moderate to severe atopic dermatitis enrolled in a phase 3 open-label extension study. J Am Acad Dermatol.

[7] Thyssen JP, de Bruin-Weller MS, Paller AS, Leshem YA, Vestergaard C, Deleuran M (2019). Conjunctivitis in atopic dermatitis patients with and without dupilumab therapy—international eczema council survey and opinion. J Eur Acad Dermatol Venereol.

[8] Treister AD, Kraff-Cooper C, Lio PA (2018). Risk factors for dupilumab-associated conjunctivitis in patients with atopic dermatitis. JAMA Dermatol.

[9] Nahum Y, Mimouni M, Livny E, Bahar I, Hodak E, Leshem YA (2020). Dupilumab-induced ocular surface disease (DIOSD) in patients with atopic dermatitis: clinical presentation, risk factors for development and outcomes of treatment with tacrolimus ointment. Br J Ophthalmol.

[10] Maudinet A, Law-Koune S, Duretz C, Lasek A, Modiano P, Tran THC (2019). Ocular surface diseases induced by dupilumab in severe atopic dermatitis. Ophthalmol Ther.

[11] Shen E, Xie K, Jwo K, Smith J, Mosaed S (2019). Dupilumab-induced follicular conjunctivitis. Ocul Immunol Inflamm.

[12] Levine RM, Tattersall IW, Gaudio PA, King BA (2018). Cicatrizing blepharoconjunctivitis occurring during dupilumab treatment and a proposed algorithm for its management. JAMA Dermatol.

[13] Paulose SA, Sherman SW, Dagi Glass LR, Suh LH (2019). Dupilumab-associated blepharoconjunctivitis. Am J Ophthalmol Case Rep..

[14] Liberman P, Shifera AS, Berkenstock M (2020). Dupilumab-associated conjunctivitis in patients with atopic dermatitis. Cornea..

[15] Barnes AC, Blandford AD, Perry JD (2017). Cicatricial ectropion in a patient treated with dupilumab. Am J Ophthalmol Case Rep.

[16] Yamane MLM, Belsito DV, Glass LR (2019). Two differing presentations of periocular dermatitis as a side effect of dupilumab for atopic dermatitis. Orbit..

[17] Zirwas MJ, Wulff K, Beckman K (2018). Lifitegrast add-on treatment for dupilumab-induced ocular surface disease (DIOSD): a novel case report. JAAD Case Rep.

[18] Guglielmetti S, Dart JK, Calder V (2010). Atopic keratoconjunctivitis and atopic dermatitis. Curr Opin Allergy Clin Immunol.

[19] de Bruin-Weller M, Graham NMH, Pirozzi G, Shumel B (2018). Could conjunctivitis in patients with atopic dermatitis treated with dupilumab be caused by colonization with Demodex and increased interleukin-17 levels?: reply from the authors. Br J Dermatol.

[20] Thyssen JP (2018). Could conjunctivitis in patients with atopic dermatitis treated with dupilumab be caused by colonization with Demodex and increased interleukin‐17 levels?. Br J Dermatol.

[21] Mennini M, Dahdah L, Fiocchi A (2017). Two phase 3 trials of dupilumab versus placebo in atopic dermatitis. N. Engl J Med.

[22] Thyssen JP, Toft PB, Halling-Overgaard AS, Gislason GH, Skov L, Egeberg A (2017). Incidence, prevalence, and risk of selected ocular disease in adults with atopic dermatitis. J Am Acad Dermatol.

[23] Bonini S (2004). Atopic keratoconjunctivitis. Allergy.

[24] Tuft SJ, Kemeny DM, Dart JK, Buckley RJ (1991). Clinical features of atopic keratoconjunctivitis. Ophthalmology..

[25] Aszodi N, Thurau S, Seegräber M, de Bruin-Weller M, Wollenberg A (2019). Management of dupilumab-associated conjunctivitis in atopic dermatitis. J Dtsch Dermatol Ges.

[26] Utine CA, Stern M, Akpek EK (2010). Clinical review: topical ophthalmic use of cyclosporin A. Ocul Immunol Inflamm.

[27] Hoy SM (2017). Ciclosporin ophthalmic emulsion 0.1%: a review in severe dry eye disease. Drugs..

[28] Sernicola A, Gattazzo I, Di Staso F, Giordano D, Capalbo A, Persechino F (2019). Treatment of refractory conjunctivitis associated to dupilumab with topical pimecrolimus applied to the eyelid skin. Dermatol Ther.

